# Antibiotic treatment regimens for bone infection after debridement: a study of 902 cases

**DOI:** 10.1186/s12891-020-03214-4

**Published:** 2020-04-07

**Authors:** Xiaohua Wang, Li Fang, Shulin Wang, Yueqi Chen, Huan Ma, Hongwen Zhao, Zhao Xie

**Affiliations:** 1 Department of Orthopaedics, First Affiliated Hospital, Third Military Medical University (Army Medical University), Gaotanyan No. 30, Chongqing, 400038 People’s Republic of China; 2 Department of Kidney, First Affiliated Hospital, Third Military Medical University (Army Medical University), Chongqing, 400038 People’s Republic of China; 3 Department of Pharmacy, First Affiliated Hospital, Third Military Medical University (Army Medical University), Chongqing, 400038 People’s Republic of China

**Keywords:** Bone infection, Short-term antibiotic, Liver damage, Renal damage

## Abstract

**Background:**

Our aim was to investigate the clinical efficacy and complications of antibiotic treatment regimens for patients with bone infection.

**Methods:**

We retrospectively analysed patients with bone infection admitted to our hospital between March 2013 and October 2018. After surgical debridement was performed, the patients were divided into three groups: IV group (intravenous antibiotics for 2 weeks); oral group (intravenous antibiotics for 2 weeks followed by oral antibiotics for 4 weeks); and rifampicin group (intravenous antibiotics for 2 weeks followed by oral antibiotics plus rifampicin for 4 weeks). The infection control rate and complications were compared among the three groups.

**Results:**

A total of 902 patients were enrolled. The infection sites included 509 tibias, 228 femurs, 32 humeri, 23 radii and ulnae, 40 calcanei, and 47 miscellaneous sites, as well as 23 multiple-site infections. After at least 6 months of follow-up, 148 (16.4%) patients had an infection recurrence. The recurrence rate of the IV group was 17.9%, which was not significantly higher than the recurrence rates of the oral group (10.1%) and rifampicin group (10.5%), *P* = 0.051. The incidence of abnormal alanine aminotransferase (ALT) levels in the IV group was 15.1%, which was lower than that in the oral group (18.0%) and rifampicin group (27.4%), *P* = 0.026. The rates of proteinuria in the three groups were 3.2, 4.5, and 9.3%, respectively, *P* = 0.020.

**Conclusions:**

After debridement of bone infection, short-term antibiotic treatment regimens might offer similar rates of infection eradication while avoiding the risk of renal and hepatic damage associated with prolonged antibiotic use.

**The level of clinical relevance:**

Stage III.

## Background

Bone infection is a common complication after bone fracture. Although modern medical advancements have brought the treatment of bone infections to a new height, it is still a difficult disease for orthopaedic doctors to treat. The commonly used treatments include conservative debridement, continuous flushing, antibiotic carrier filling, and others. To achieve the desired results, clinicians frequently use parenteral antibiotic therapy at a high dose for prolonged periods [1]. However, intravenous therapy carries substantial risks, and it is inconvenient for patients [2]. In particular, long-term intravenous antibiotic therapy may cause complications such as catheter-related bloodstream infections and thrombosis [3]. Currently, the mainstay treatment method is debridement followed by systemic antibiotic therapy, which commonly includes intravenous infusion for 2 weeks followed by oral antibiotics for 4–6 weeks [4, 5]. However, there is no evidence that antibiotic therapy for 4–6 weeks leads to improved outcomes compared with shorter regimens [1]. Moreover, the long-term application of antibiotics may lead to adverse liver and kidney reactions [6]. The route and duration of antibiotics after debridement is important for infection eradication. Scholars are trying to reduce the use of antibiotics, but there is currently no accepted plan. In this study, we explored the use of a 2-week IV antibiotics regimen in the treatment of bone infection, and the overall efficacy was adequate.

## Patients and methods

After receiving approval from our institutional review board, we retrospectively analysed patients with bone infection admitted to our hospital between March 2013 and October 2018. The diagnosis of bone infection was made based on the presence of local bone pain and swelling during examination, imaging procedures, microbiological and histopathological examinations, and laboratory studies (such as biopsy, which is the gold standard, or deep tissue culture) [7]. The inclusion criteria were as follows: bone infection; treatment with debridement and subsequent filling with antibiotic cement; and use of postoperative systemic antibiotics. The exclusion criteria were as follows: diabetic foot infection; no surgical treatment; autoimmune disease and generalized vascular insufficiency; incomplete follow-up data; preoperative liver or kidney dysfunction; or bone graft performed within 6 months after debridement.

### Treatment methods

In all patients, debridement was performed, and antibiotic cement was implanted. After the cement was removed (following eradication of the infection), bone grafts were performed to repair the bone defects. In this study, only the infection control process was considered; the process of repairing the bone defect was not evaluated. During the operation, sequestrum and necrotic tissue were removed, and bacteria were identified by a drug susceptibility test. After debridement, bone defects were filled with antibiotic polymethyl methacrylate(PMMA) cement (Heraeus, Germany). The appropriate fixation method (plate internal fixation, plate external fixation, no fixation) was chosen considering the Cierny-Mader classification, and negative pressure drainage was allowed for 10 to 12 days.

The patients were divided into three groups according to whether oral antibiotics were taken after surgery: an IV group (intravenous antibiotics for 2 weeks); an oral group (intravenous antibiotics for 2 weeks followed by oral antibiotics for 4 weeks); and a rifampicin group (intravenous antibiotics for 2 weeks followed by oral antibiotics plus rifampicin for 4 weeks). There were no significant differences in any of the following general data: fixation method used; Cierny-Mader classification; or bacterial infection. The bacterial infection and the types and doses of antibiotics used are shown in Table 1.

**Table 1:** The bacteria and the antibiotics we used

Bacteria	Intravenous antibiotics		Oral antibiotics	
	Adults	Pediatrics	Adults	Pediatrics
MSSA/MSSE	Cefazolin:2 g q8h. Cefuroxime:1.5 g q8h. Ceftriaxone:2 g q12h. Levofloxacin:600 mg qd, Moxifloxacin:400 mg qd	Cefazolin:50-100 mg/(kg.d) q8h. Cefuroxime: 30-100 mg/(kg.d) q8h. Ceftriaxone:20-80 mg/(kg.d) qd	Levofloxacin:500 mg qd, Moxifloxacin:400 mg qd) . Cefuroxime:500 mg bid	Cefuroxime: 10 mg/Kg, bid
MRSA/MRSE	Vancomycin:15~20 mg/Kg q8~12h. Linezolid:600 mg q12h	Vancomycin:40 mg/(kg.d) 2 to 4 times/d. Linezolid: 10 mg/kg q8h	Linezolid:600 mg q12h. Fluoroquinolone.	Linezolid: 10 mg/kg q8h
*P.aeruginosa/E.coli/E.cloaca/K. pneumoniae*	Ceftazidime:2 g q8h/Cefepime:2 g q12h/PiperacillinTazobactam:4.5 g q8h /Levofloxacin;500 mg qd/Amikacin:15 mg/Kg qd (usually used in combination)	Ceftazidime:30-100 mg/(kg.d) q8h. Cefepime:40 mg/Kg q12h. PiperacillinTazobactam:112.5 mg/Kg q8h	Levofloxacin: 500 mg qd	No oral
Others	According to drug sensitivity	According to drug sensitivity	According to drug sensitivity	According to drug sensitivity
Negative	Ceftazidime / Cefepime / Fluoroquinolone	Ceftriaxone 20-80 mg/(kg.d) qd	Fluoroquinolone	Cefuroxime: 10 mg/Kg, bid

MSSA/MSSE: Methicillin-susceptible *Staphylococcus aureus/ epidermidis*. The dosage is suitable for patients with no serious liver and renal dysfunction

### Postoperative management and follow-up

All patients were examined by laboratory tests and urine protein tests on the 1st, 7th, and 14th days after surgery. Subsequently, the same examinations were repeated weekly for the oral group and rifampicin group, and urine protein was repeatedly examined until the 6th week. The infection control and recurrence criteria are as follows [6]: an apparent cure was defined as the absence of signs or symptoms of osteomyelitis for at least 6 months after the cessation of antimicrobial therapy. A relapse was defined as an infection occurring at the same site from which it was apparently eliminated, requiring specific treatment with antibiotics or surgery. ALT and aspartic transaminase (AST) values greater than 40 U/L and creatinine (Cr) values greater than 104 μmol/L were defined as abnormal.

#### Statistics

One-way analysis of variance (or the rank sum test, where relevant) was used to compare measurement data (recurrent time). Pearson’s chi-square test was used to compare enumeration data (ALT, AST, and Cr levels, proteinuria, infection control rate). A *P* value below 0.05 was considered significant.

## Results

A total of 902 patients were enrolled in this study. There were 717 males and 185 females with an average age of 40.25 (4–76) years. The mean duration of infection before admittance to the hospital was 70.7 months (14 days to 720 months). The infection sites included 509 tibias, 228 femurs, 32 humeri, 23 radii and ulnae, 40 calcanei, and 47 miscellaneous sites, as well as 23 multiple-site infections. There were 666 cases of post-traumatic infection and 236 cases of haematogenous osteomyelitis. Bacteria were isolated from 640 (71%) patients. Of these patients, 307 (48.0%) were infected with *Staphylococcus aureus* (including 81 with MRSA), 56 (8.8%) with *Pseudomonas aeruginosa*, 41 (6.4%) with *Escherichia coli*, 33 (5.2%) with *Enterobacter cloaca*, 39 (6.0%) with *Staphylococcus epidermidis*, 25 (3.9%) with *Klebsiella pneumoniae,* 64 (10%) with other bacteria, 75 (11.7%) with more than one bacteria, and the remaining 262 cases were negative.

There were 148 (16.4%) recurrent infections within the six-month follow-up period, with an average recurrence time of 63.0 ± 4.8 (4–176) days. The recurrence rate of the IV group was 17.9% (130/727), which was not significantly higher than that of the oral group (10.1%, 9/89) and the rifampicin group (10.5%, 9/86); *P* = 0.051. The recurrence times of the three groups were 62.37 ± 6.93, 58.56 ± 13.44, and 76.33 ± 17.48 days, respectively; *P* = 0.851. There were 727 patients in the IV group, of which 130 patients relapsed. The highest recurrence rate was observed among patients with *P. aeruginosa* infections, who had a recurrence rate of 40.4% (19/47), while the recurrence rate of patients with no isolated bacteria was 8.1% (19/236); *P* < 0.01.

The rate of abnormal ALT levels in the IV group was 15.1%, which was lower than that of the oral group (18.0%) and rifampicin group (27.4%); *P* = 0.026. The rates of proteinuria in the three groups were 3.2, 4.5, and 9.3%, respectively; *P* = 0.020. There were no significant differences in the rates of abnormal AST (*P* = 0.798) and Cr (*P* = 0.620) levels among the three groups. The results are shown in Table 2.

**Table 2:** Comparisons of general information, clinical efficacy of the three groups

Events	IV group	Oral group	Rifampicin group	P Value
Number	727	89	86	–
Sex ratio (male/female)	3.9 (578/149)	4.6 (73/16)	3.3 (66/20)	0.688
Average age(years)	37.11 ± 1.62	40.30 ± 3.22	38.91 ± 2.54	0.130
Average duration of infection(months)	70.3 ± 6.5	68.1 ± 11.8	75.9 ± 8.1	0.255
Type of infection (posttraumatic/hematologic)	(541/186)	(67/22)	(58/28)	0.360
Recurrence rate of infection	17.9%(130/727)	10.1%(9/89)	10.5%(9/86)	0.051
Recurrence time(days)	62.37 ± 6.93	58.56 ± 13.44	76.33 ± 17.48	0.851
Abnormal rate of ALT	15.1%(110/727)	18.0%(16/89)	27.9%(24/86)	**0.026**
Abnormal rate of AST	16.4%(119/727)	14.6%(13/89)	13.9%(12/86)	0.798
Abnormal rate of Cr	1.1%(8/727)	1.1%(1/89)	2.3%(2/86)	0.620
Positive rates of proteinuria	3.2%(23/727)	4.5%(4/89)	9.3%(8/86)	**0.020**

*ALT* Alanine aminotransferase, *AST* Aspartic transaminase, *Cr* Creatinine

## Discussion

Treatment of bone infection is a difficult procedure. At present, the overall treatment strategy includes conservative treatment, palliative debridement, en bloc resection, and amputation, but there is a lack of uniform treatment standards. Surgery is the usual method chosen by clinicians, but not all cases require surgical debridement [1], and many infections can be effectively treated with oral therapy [8–10]. Hamed [11] reported that intravenous antibiotics (6 weeks) and oral suppression therapy were administered for cases of PJI infection, with reliable clinical efficacy. Research has shown that drug therapy alone has an average success rate of 71.1%, while surgical treatment alone has an average success rate of 88.9% [12]. Systemic intravenous antibiotics after debridement can achieve a rapid reduction in the bacterial load at the site of infection [13]. Therefore, clinicians are more inclined to use surgical treatment combined with antibiotic treatment to stop the infection.

According to previous studies, the optimal treatment of acute haematogenous osteomyelitis involves intravenous antibiotics for several weeks and then oral antibiotics until the symptoms and signs are alleviated [14, 15]. However, for chronic bone infection, debridement is often the first choice. Although research has shown that oral and parenteral therapies achieve similar success rates [1], clinicians are accustomed to considering the use of intravenous antibiotics [16, 17], followed by oral antibiotics for several weeks, during hospitalizations. It has been reported that the use of intravenous antibiotics for less than 1 week has no significant effect on prognosis [18–20]. However, only limited data comparing intravenous antibiotics and oral antibiotics or concerning proper treatment durations are available. We applied debridement plus systemic antibiotic treatment. Most patients (80.6%) were treated with intravenous antibiotics for only 2 weeks, although long-term antibiotic use has achieved good results in previous reports. Our results showed that the recurrence rate of the IV group was not significantly different from that of the other two groups, and the overall infection control rate was 83.6%. The infection control rate is comparable to that of previous reports, providing support for the treatment of these patients with short-term systemic antibiotic regimens following debridement.

Multiple and refractory bacterial infections are common in bone infections, especially chronic infections, so combined antibiotic treatments are commonly applied in the clinic. The most common oral combination drug is rifampicin, which has a bactericidal effect on multiple gram-positive and gram-negative bacteria [17]. Rifampicin can achieve high intracellular levels and is capable of penetrating bacterial biofilms [21, 22]. However, it is not advisable to only apply rifampicin for anti-infection treatment because resistance can quickly develop [5]. Thus, rifampicin should be started after the bacterial load is reduced by surgery and when the wound is dry [23, 24]. In this study, 307 (48.0%) patients were infected with *Staphylococcus aureus* and 56 (8.8%) with *Pseudomonas aeruginosa,* accounting for more than 50% of the isolated bacteria, so levofloxacin was applied more often in our clinic. Levofloxacin alone was unable to eradicate methicillin-susceptible *S. aureus* [25, 26]. The classic antibiotic combination for bone infections caused by *Staphylococcus aureus* and *P. aeruginosa* is levofloxacin plus rifampicin.

It is difficult to assess how long it will take for an infection to clear following the treatment of bone infection. Urania Rappo [27] evaluated the efficacy of infection after 6 weeks of treatment. Daver NG [6] indicated that the infection is apparently cured if it does not recur within 6 months. We evaluated infection control at 6 months after the operation. Although this timing may miss some cases of recurrence, our statistical analysis shows that recurrence occurs within 2 months in most cases.

Although rifampicin is rarely used alone in the treatment of bone infections, when this drug used in combination with other antibiotics, rifampicin can increase the treatment outcomes of other antibiotics. Rifampicin diffuses very well within bone tissue and bacterial biofilms [28] and is a valuable treatment option. However, rifampicin often elicits a hepatotoxic response and leads to gastroenteropathy. Our results indicated that the rate of abnormal ALT levels in the rifampicin group was significantly higher than that in the other groups, suggesting that postoperative combinations including rifampicin may lead to liver damage. In addition, Sahoko [29] reported that rifampicin can cause acute renal damage. Our results show that the overall complication rate was acceptable in the three groups. The recurrence rate of the IV group was not significantly different from that of the other two groups. Moreover, the oral group and rifampicin group had higher ALT levels and proteinuria rates, and the abnormal levels were probably caused by the additional oral therapies. Although the proteinuria rate in the rifampicin group was the highest, the rate of abnormal creatinine levels was not significantly different, suggesting that the additional rifampicin therapies may cause early or mild renal damage.

There are many strengths in this article, including the large sample size and its use of standard techniques. However, there are several drawbacks. First, although our results showed that there was no significant difference in the general data, including the type of bacterial infection and fixation method, the heterogeneity within the cohort may have affected the results. Second, the abnormal ALT and proteinuria levels suggested possible liver and renal damage, but the extent of the damage was not determined. Third, recurrence is common in bone infection, and the long-term recurrence rate for patients (after the 6-month follow-up) is unknown.

## Conclusions

After debridement of bone infection, short-term antibiotic treatment regimens might offer similar rates of infection eradication while avoiding the risk of renal and hepatic damage associated with prolonged antibiotic use.

## Data Availability

We are unable to share the raw data because ethical approval was not obtained for data sharing. In addition, all data are presented in the Tables.

## Abbreviations

ALT: Alanine aminotransferase
AST: Aspartic transaminase
Cr: Creatinine
MSSA: Methicillin-susceptible *Staphylococcus aureus*
MSSE: Methicillin-susceptible epidermidis
